# The Relationship Between Executive Dysfunction and Thought–Action Fusion in Obsessive–Compulsive Disorder

**DOI:** 10.1192/j.eurpsy.2026.11601

**Published:** 2026-07-31

**Authors:** I. Otrokh, I. Otrokh

**Affiliations:** Neuropsychology, Taras Shevchenko National University of Kyiv, Kyiv, Ukraine

## Abstract

**Introduction:**

Obsessive–compulsive disorder (OCD) involves intrusive thoughts (obsessions) and rituals (compulsions) performed to reduce distress. Recent studies highlight the role of executive dysfunction in sustaining obsessive–compulsive symptoms. Another key cognitive bias is thought–action fusion (TAF), where simply having a thought is regarded as equivalent to carrying it out. Although both executive dysfunction and thought–action fusion are recognized as important features of OCD, their relationship remains underexplored.

**Objectives:**

This study aimed to examine the relationship between executive dysfunction and thought–action fusion in adults diagnosed with obsessive-compulsive disorder.

**Methods:**

The study included 81 participants: 27 adults with an ICD-11 diagnosis of OCD (M = 29.9, SD = 7.87) and 54 controls without psychiatric history (M = 20.7, SD = 2.44). Instruments included the Yale–Brown Obsessive Compulsive Scale (CY-BOCS), Stroop Test, Thought–Action Fusion Scale–Revised (TAFS-R), and the Executive Skills Questionnaire–Adult Version (ESQ–A). Data were analyzed using Pearson correlations and multivariate analysis of covariance (MANCOVA) in Jamovi.

**Results:**

The study tested whether dimensions of thought–action fusion (moral, likelihood-other, likelihood-self ) mediated the relationship between executive dysfunction and obsessive–compulsive symptoms. The analysis revealed a significant main effect of group membership on executive function scores, F(13, 63) = 37.238, p < .001, η² = .885. Regarding the TAF dimensions, moral TAF and TAF likelihood-other were not significant predictors, whereas TAF likelihood-self (fusion regarding one’s own thoughts) was statistically significant, V = .3105, F(13, 63) = 2.183, p = .021, η² = .311. Higher endorsement of TAF likelihood-self was linked to poorer metacognition, reduced flexibility, and impaired inhibition. Group differences remained significant for attention (F(1, 75) = 11.747, p < .001), planning (F(1, 75) = 9.158, p = .003), flexibility (F(1, 75) = 8.365, p = .005), and Stroop performance (F(1, 75) = 365.871, p < .001). These findings suggest that biased appraisals of one’s own thoughts as dangerous or morally significant may contribute to executive dysfunction in OCD, particularly in domains of flexibility and inhibition.

**Conclusions:**

The findings suggest that executive dysfunction in OCD cannot be explained solely by symptom severity but is closely linked to maladaptive cognitive biases, particularly thought–action fusion of one’s own thoughts. Biased appraisals of personal intrusive thoughts appear to exacerbate deficits in metacognition, cognitive flexibility, and inhibitory control. This suggests that targeting both executive processes and cognitive distortions may be essential in developing more effective interventions for OCD. Future research should explore their role in treatment response.

**Disclosure of Interest:**

None Declared

